# Prof. Liebig’s Soluble Food in the Treatment of Diseases of the Digestive Organs

**Published:** 1872-09-15

**Authors:** A. H. Salisbury

**Affiliations:** Mazomanie, Wis.


### Domestic Correspondence.

### PROF. LIEBIG'S SOLUBLE FOOD IN THE TREATMENT OF DISEASES OF THE DIGESTIVE ORGANS.

I wish to call the attention of the profession to an article of food, the merits of which I believe are not generally known. I have had (and what practitioner has not) a number of cases of gastric and intestinal disturbance which seemed to resist all forms of treatment.

My little patients from three months to two years old were unable to digest food of the most simple kind.

Milk, arrowroot, and all forms of starch, beef tea, animal broths prepared in the most careful manner, were alike rejected by the stomach, or passed the bowels undigested.

The thin mucus discharges, sometimes mingled with blood, were very frequent, and, in some cases, attended by tenesmus.

Drugs were of little use. Opium, astringents, bismuth, chalk, pepsin, mercury, were equally useless.

Meanwhile the little sufferers were rapidly wasting away, actually starving to death for want of some food which the diseased organs could assimilate.

With but little faith I at last ordered "Liebig's Soluble Food." Under this treatment the first child began to improve without medicine of any kind. I gave it to the others. They all improved.

A brother practitioner pronounced the food *sorghum molasses*, but nevertheless gave it a trial. His patients improved. In every case the irritability of the stomach ceased, and the discharges became less frequent and more natural.

The result is that thus far we have not lost a case of this kind, though of several we had no hope. The children relish it, in fact cry for it while they refuse all other food.

It is recommended to be prepared with equal parts of skimmed milk and water. I have found it better, in these cases, to use water alone.

The article in question is prepared from Prof. Liebig's prescription by J. Paul Lerhe, of Dresden. If the experience of others is the same as mine, the great chemist has supplied a want long felt, in giving us this preparation.

A. H. Salisbury, M. D.

Mazomanie, Wis.

